# Botulinum Toxin in Chronic Lateral Epicondylitis, from Tendon to Muscle Approach—A Review

**DOI:** 10.3390/life14040528

**Published:** 2024-04-20

**Authors:** Daniela Poenaru, Miruna Ioana Sandulescu, Claudia-Gabriela Potcovaru, Delia Cinteza

**Affiliations:** Rehabilitation Department, Carol Davila University of Medicine, 050474 Bucharest, Romania; miruna.sandulescu@drd.umfcd.ro (M.I.S.); claudia-gabriela.potcovaru@drd.umfcd.ro (C.-G.P.); delia.cinteza@umfcd.ro (D.C.)

**Keywords:** botulinum toxin, lateral epicondylitis, intramuscular injection, intratendinous injection

## Abstract

Background: Chronic lateral epicondylitis challenges the therapeutical approach; underlying mechanisms are incompletely understood; neuropathic pain and central and peripheral sensitization may explain the fact that botulinum toxin has been found to play a role in pain and function management. Methods: We searched the literature for MeSH terms: lateral epicondylitis or synonyms and botulinum toxin. Results: We found 14 papers containing trials on botulinum toxin injection into the tendon or into the extensor muscles (specifically, extensor carpi radialis brevis and extensor communis digitorum). We followed the administration pathways, doses, timing, and side effects. Conclusions: With a chronic course, the focus of the therapy shifts from the afflicted tendon to the inserting muscles, as muscle contracture may create a vicious loop to perpetuate and aggravate the disease. Doses, timing, and side effects are discussed.

## 1. Introduction

Lateral epicondylitis (LE) has many names in the literature: common extensor tendinopathy, radial epicondylitis, tennis elbow, radial epicondylalgia. Despite its terminology, it is a rather non-inflammatory, overuse, degenerative condition, the most common elbow affliction, with a prevalence in the general population of 1–3% and in people who perform physically demanding work of 15% [1]. The affected structure is the common extensor tendon for the following muscles: extensor carpi radialis brevis (ECRB), extensor digitorum communis (EDC), extensor carpi ulnaris, and extensor digiti minimi, the first two muscles being the most involved. As a self-limitative disease, 83% of patients recover after 12 months with rest and pain control pills [2]. Chronic evolution or recurrence require more active therapies: splinting, corticosteroid injections, prolotherapy, physical agents, platelet-rich plasma, etc.

The primary cause of LE is the contractile overload of the tendon, producing repetitive stress. Furthermore, researchers demonstrated a low blood flow within the ECRB, presuming that impaired muscle microcirculation and reduced oxygen supply contribute to the symptoms [3]. Notably, recent studies highlighted peripheral and central sensitization as mechanisms of pain in chronic unilateral LE, similar to many local pain syndromes (myofascial syndrome, temporomandibular disease, whiplash, repetitive strain injury, nonspecific arm pain, chronic tension type headache, low back pain, knee osteoarthritis, and unilateral shoulder pain) [4,5]. These mechanisms may be responsible for the failure of conservative and surgical therapies.

In the context of recurrence or chronicity of LE, new conservative therapies are to be researched. Extracorporeal shock wave therapy is extensively cited in studies on human and animal models, as well as platelet-rich plasma (PRP) injections, a regenerative therapy that is gaining researchers’ and clinicians’ attention [6].

Botulinum toxin (BTX), an exotoxin of Clostridium botulinum, acts as an inhibitor of acetylcholine release in the neuromuscular junction, with a transitory effect. It is known to also have an analgesic effect, inhibiting the release of neurotransmitters and neuropeptides such as glutamate, calcitonin gene-related peptide, and substance P. Both pathways are investigated in the treatment of LE: the paralyzing effect is followed by reduced burden on the tendon, allowing self-repair and a direct analgesic effect on the tendon. Additionally, muscle relaxation increases intramuscular blood flow, improves aerobic metabolism, lowers lactate concentration, and reduces pain. Additional effects of BTX may count on the inhibition of the release of neurotransmitters from peripheral nerve endings, reducing the afferent input to the spinal cord and, consequently, peripheral sensitization that, indirectly, reduces central sensitization. Central sensitization may also be interrupted by the retrograde axonal transport of the toxin along the branches of nociceptive neurons [7,8,9].

There are seven distinct serotypes of BTX (from A to G), with types A and B used in clinical practice. The type A toxin (BTX-A) has an endopeptidase light chain that, in the presence of zinc, deactivates the synaptosomal-associated protein with a molecular mass of 25 kDn (SNAP 25) within the cell membrane. Recent identification of the SNAP 25 on sensory neurons may account for the antinociceptive effect of BTX-A [8]. BTX-A is commercially available in four forms: onabotulinumtoxin A (Botox, Allergan, Irvine, CA, USA), abobotulinumtoxin A (Dysport, Ipsen, Cherry Valley, IL, USA), and incobotulinumtoxin A (Xeomin, Merz, Frankfurt, Germany) available in the United States, and a Chinese toxin, Prosigne (Lanzhou Institute, Lanzhou, China). BTX-B is available in rimabotulinumtoxin B (marketed as Myobloc in the United States and Neurobloc in Europe; Solstice). These toxins have different units and different potencies.

According to the above-mentioned mechanisms of action of BTX-A, we identified two main therapeutical approaches for LE: intratendinous injection (based on local analgesic effect) and intramuscular injection (based on relaxation effect). In fact, it is a unique situation in which tendinopathy is treated either by a direct approach or by an “at distance” approach, targeting the connecting muscle.

The aim of this study is to highlight the indications, timing, doses, modality of administration, and adverse effects of BTX-A. We assumed the hypothesis that botulinum toxin injections are important third-line options for refractory or chronic LE cases.

## 2. Materials and Methods

We searched PubMed, MEDLINE, Google Scholar, EMBASE, and PEDro, starting from inception up to 2023, following the MeSH terms lateral epicondylitis OR radial epicondylitis OR common extensor tendinopathy OR tennis elbow AND botulinum toxin. Inclusion criteria were clinical trials on human subjects published up to December 2023. Two authors independently performed this task, and they gathered 1820 titles. After excluding duplicates, 1570 titles were selected, and titles were analyzed. From the remaining 87 relevant titles, the authors independently read the abstracts, excluded the reviews, and retained 14 clinical studies [10,11,12,13,14,15,16,17,18,19,20,21,22,23]. There were 63 articles excluded for being studies on animal models or on cultured cells. All disagreements were solved by discussion with all team members. All studies but one ([11], written in German) were written in English. Our paper aims to identify the indications of BTX-A for LE, the targeted structures, the most valuable injection technique, the dosage, timing, and the side effects.

## 3. Results

**Figure 1:**
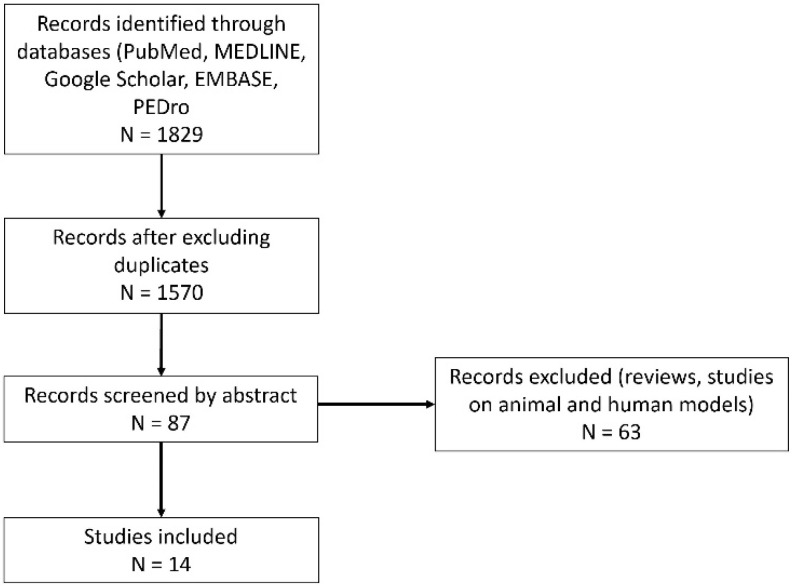
Selection process of the literature.

Of the final 14 studies, 2 studies focused on intratendinous administration [13,22], whereas the remaining 12 studies focused on intramuscular administration. All but one [18] included patients with chronic LE. In one study [13], patients with chronic LE were administered BTX as a first line of therapy, whereas twelve papers reported BTX administration on chronic LE with failure of conservative therapies (Figure 1 and Table 1).

**Figure 2:**
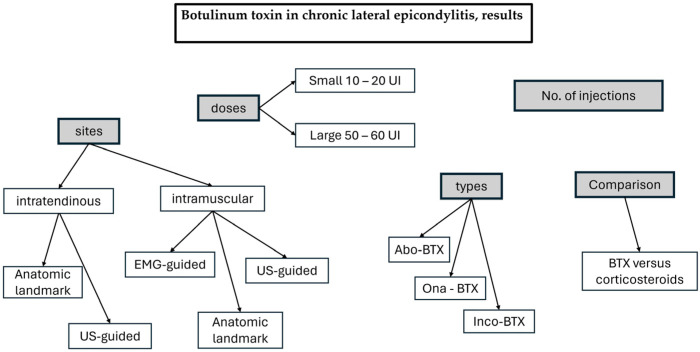
The main topics in the Results section.

The following results are presented in Figure 2 to underline the main issues from the analysis.

### 3.1. Intratendinous Injection

Intratendinous injections were performed based on either anatomical landmarks or ultrasound guidance. In an anatomical landmark study, the authors described the point of needle insertion 1 cm distal to the lateral epicondyle, into the subcutaneous tissue and muscle, toward the tender spot. However, we presume that the targeted structure was the tendon rather than the muscle, as the length of the common extensor tendon is about 1.8 cm [24]. Patient selection included at least 3-month-old epicondylitis with no previous local injection therapy (corticosteroid or acupuncture). Compared to the placebo, 60 UI abobotulinumtoxin A produced significant pain reduction at 4 and 8 weeks. Side effects were weakness of finger extension and paresis of digits in 33% of patients at 4 weeks and in 7% at 12 weeks, with one patient reporting interference with job activity, most probably due to accidental leakage of the toxin toward the muscle belly [13].

The peppering technique in the tendon under ultrasound guidance was used to comparatively study small doses (10 UI) versus large doses (50 UI) of BTX-A for chronic LE. Both doses produced significant pain reduction and grip strength improvement up to 6 months, with better results for the higher dose. The 50 UI dose elicited more extensor weakness versus the small 10 UI dose (20% vs. 3%), most probably due to toxin leakage. The authors stressed that the results were due to the local analgesic effect rather than the paralytic one, as the grip strength increased in their study instead of decreasing as noted in the studies with intramuscular injections [22].

### 3.2. Intramuscular Administration

Despite the fact that LE is a tendinopathy, intramuscular administration of BTX was the topic of a great number of papers. The mark of correct intramuscular injection was considered the presence of the extensor lag, i.e., the inability to fully extend a digit actively. Some authors even repeated the injection until they obtained an extensor lag to document proper targeting [11]. As the effect of BTX is transitory, an extensor lag of about 3 months would be expected in the studies. The most targeted finger was the third, and some working activities may be impaired. It may be a contraindication for this therapy.

In 1997, the first open study included 14 patients with treatment-resistant chronic LE. The authors performed electromyographic-guided injection BTX-A (20–40 UI) into the extensor digitorum communis III and IV muscle. The rationale behind this approach was the clinical observation that the most painful test was the resisted extension of digits III and IV; therefore, they had to be targeted to obtain paresis. If paresis was not obtained after one injection, a second dose was administered after one month. All but one patient reported pain relief in an interval between 2 weeks and 1 month and maintenance of the effect at 6–8 months in a significant proportion. Pain reduction persisted beyond the paralyzing effect. No side effects were noted, apart from the expected finger extensor weakness that disappeared after 3–4 months [10].

The same technique was used in a pilot randomized study on a BTX-A administration group (30–40 UI into ECRB) versus an operative group (Hohmann procedure), both therapies aiming to relax the affected tendon. When the first dose did not produce paresis, a second greater dose (50 UI) was injected at 6 weeks. At 3 months, there was a significant difference for sick leave in favor of the operative group; the difference disappeared at 6, 12 and 24 months. At 3 and 6 months, the operative group had a greater extension deficit but not at 12 and 24 months, and there was no difference between pain scores in both groups at all moments. Overall scores were comparable between groups. Patients that did not achieve sufficient paresis were recommended for surgery, but the postoperative evolution was negative, suggesting a more complex pain mechanism as the central chronic pain [11].

Based on the observation that persistent ECRB muscle contraction inhibits local microcirculation, decreases oxygen supply, and promotes local anaerobiosis [3], researchers observed a reversal of these phenomena and significant pain relief at 3 and 12 months after BTX-A injection of the ECRB. Functional improvement was also noted but not to a significant degree. Grip strength significantly declined at 3 months and increased at 12 months. The doses were 1 UI/kg body weight onabotulinumtoxin A with an upper limit of 100 UI/muscle. The ECRB was targeted by EMG and the injection was performed at a point two fingerbreadths distal to the lateral epicondyle. The accuracy of injection was also confirmed by the transitory reduction in muscle strength 3 months later and normalization at 12 months [16].

Another randomized, placebo-controlled, double-blinded study confirmed significant pain and quality of life (self-assessment) improvement at 30 and 90 days after 40 UI aboBTX-A injection into the ECRB identified with EMG stimulation. Since the targeted structure was the ECRB, there were no side effects, especially no extensor lag and no grip strength alteration [20].

A small prospective, observational pilot study included 16 patients who received 60 UI on an anatomical landmark: approximately 3 to 4 cm distal to the tender epicondyle, with infiltration of the muscle at two locations; the second location was injected after partial withdrawal of the needle and rotating it in the horizontal plane. The targeted structure was certainly the group of extensor muscles, with no attempt to individualize them. The improvement in pain scores and subjective self-assessment were significant at 2 weeks and maintained up to 2 years. The side effects (weakness of third finger extension) were significant in a small percentage and slowly returned to normal. Grip strength decreased at 2 and 6 weeks and increased at 10 and 14 weeks [12]. Later, using the same technique (landmark and doses), the same authors published an extensive, prospective, placebo-controlled, double-blinded multicentric study on 132 patients that confirmed significant improvement in pain scores at all moments (up to 18 weeks) and in subjective evaluation starting from the 6th week. Grip strength did not differ from the placebo at any moment [15].

Hayton et al. published the results of a double-blinded, placebo-controlled pilot study on 40 patients that received a 50 UI onaBTX-A injection on an anatomical landmark (5 cm distal to the area of maximal tenderness at the lateral epicondyle, in line with the middle of the wrist, deep into the forearm fascia). They found no significant differences in grip strength, pain, and quality of life at 3 months for the study group or between the study group and the placebo group. A transient extensor lag was noticed in about 7% of the patients, but it was not unbearable. However, in particular situations, it might interfere with functionality at work [14].

Other researchers used a different anatomical landmark, assuming that the motor nerve branch enters the EDC and ECRB muscles at a distance of 33% of the forearm length from the lateral epicondyle. In a randomized, placebo-controlled trial, 60 UI BTX-A produced significant improvements in pain at rest and at maximum pinch up to 16 weeks. There was an insignificant and transitory reduction in the grip strength at 4 and 8 weeks. Patients reported the occurrence of an extensor lag for the third and fourth fingers, which interfered with current activity and resolved in the end of the study [17].

For a more specific muscle targeting, Galvan Ruiz et al. evaluated clinically each muscle from the common extensor tendon and injected selectively under sonographic control the afflicted bellies with a specific amount of incoBTX-A, according to muscle size, with a maximum dose of 80 UI/patient. Overall, 50% of the patients received injections into more than one muscle. The most frequent adverse effect was the expected weakness of the third finger, present up to 3 months, affecting the working capacity of patients (to be considered when proposing the therapy). Pain and function improved at one month after treatment and were maintained at 6 months (when the paralyzing effect had disappeared). The authors performed a stratification of the patients according to the intensity of the initial pain (VAS). In the group with a VAS score under 6, improvements in pain and function were higher (47% and 50%) than in the group with a VAS ≥ 6 (37% and 38%). In the latter group, altered pain processing and central sensitization in the chronic musculoskeletal condition may be responsible for these results. Another aspect underlined by the study was that 12.5% of patients required a second dose due to a positive effect but of short duration [21].

### 3.3. How Many Injections to Obtain Pain Free Evolution?

An open study investigated patients with chronic LE and ECRB injection (40 UI aboBTX-A) under EMG guidance over a one-year timeframe. Researchers noticed that after one injection, 44% of patients were satisfied and 40% asked for a second injection. After a second injection, 90% of patients were satisfied. Only 2% asked for a third injection. Overall, after one or two injections, the rate of success was 80%, underlying the cumulative effect of repeated administration. Due to the targeted injection into the ECRB, no weakness of finger extension as a side effect was noted [23].

### 3.4. Comparative Drug-Controlled Studies

A prospective, randomized, double-blinded pilot study compared three groups of patients receiving 20 UI onaBTX-A, 40 mg triamcinolone into the tendon (1 cm distal to the lateral epicondyle) or into the muscle (ECRB or EDC). Injections were performed on an anatomical landmark for the enthesis 1 cm distal to the lateral epicondyle and for the extensor muscles at the most tender point. At 4 weeks, there were significant improvements in pain and function for the intratendinous administration (BTX-A or corticosteroid) versus intramuscular administration. At 8, 12, and 16 weeks, there were no differences between the three groups. Intramuscular injection was followed by a transitory grip strength reduction at 4 weeks, which recovered at 8 and 12 weeks. It is worth mentioning that the cost of BTX-A injection is higher than the cost of corticosteroid injection; however, the rationale behind the study was the necessity of finding an alternative to corticosteroid injection due to its important rate of recurrence and other side effects [19].

Comparing intramuscular injection of 50 UI onaBTX-A or 40 mg triamcinolone in acute or subacute LE, researchers reported pain relief at 4 weeks for both groups, with a better value for the corticosteroid group. At 8 and 12 weeks, the improvement continued, but the differences between the groups were not significant. Grip strength diminished in the BTX-A group (as expected) and increased in the corticosteroid group at 4 and 8 weeks; the difference was not significant at 12 weeks. Quality of life increased in both groups without significant differences. Injection was administered to the ECRB muscle near the common origin of the wrist and finger extensors of the affected elbow, with the needle first inserted into the subcutaneous layer and then pushed further into the ECRB. Localization of needle tip in the ECRB was confirmed by palpation during resisted wrist extension [18].

## 4. Discussion

BTX-A represents an approved therapy for stroke spasticity, improving the life quality and assisting patients in achieving their goals [25]. Extending its use to chronic and recurrent tendinopathies, such as LE, with subsequent disability, is a challenge for researchers.

BTX-A is a therapeutical approach for chronic LE that failed to respond to conservative measures. Only one study used BTX-A on acute and subacute LE [18].

Two biological mechanisms sustain this indication: the temporal paralysis of extensor muscles to relax the tendon and allow healing and the release of cellular mediators (including substance P, calcitonin gene-related peptide, glutamate, and bradykinin) to reduce pain perception. Some other mechanisms may play a role, such as interference with peripheral and central sensitization associated with chronic pain.

In two papers, BTX-A was injected into the tendon, either on an anatomical landmark or under ultrasound guidance [13,22]. Pain relief was significant in the short and long term, up to 2 years. Mild weakness of finger extension was noted in 20–33% of patients, with a reversible course after 3 months, most probably due to toxin leakage.

As the disease evolves into a chronic stage, the focus of the therapy changes from the tendon to the muscle. In the acute cases, the targeted structure was the tendon. In the chronic cases, researchers targeted the corresponding muscles, as documented in the 12 studied papers. The rationale behind this approach resided in the occurrence of a vicious circle, tendon pain–muscle contraction–tensile burden on the tendon further eliciting pain. The presence of active myofascial trigger points within the forearm muscles may explain the recommendation for BTX, as studies have shown that botulinum toxin is effective [1]. In the acute phase, tendon pain is the primary target; in the chronic phase, the muscle becomes the target.

Of the four muscles that originate from the common extensor tendon, the ECRB and ECD are the most studied. They were injected together or separately, on anatomical landmarks, and guided by EMG or ultrasound. The anatomical landmarks varied throughout the trials, from 3 to 5 cm distal to the lateral epicondyle, right into the tender spot, to the first third of the forearm length. With this technique, there was no precise localization of one or another muscle; they were injected all together. ECD injection may be followed by weakness of finger extension, particularly the third finger. Paresis is reversible, generally after 3 months and, in most cases, was associated with mild and tolerable impairment. When the patients were performing tasks that required hand abilities, this therapy might have been contraindicated. Meanwhile, paresis of the third finger was considered proof that the injection was properly performed; the lack of weakness might be an indicator of a bad prognosis for surgery [11] or might require another injection to obtain the effect [11,21,23].

EMG or ultrasound guidance ensured that a targeted injection was made into the selected muscle. Four trials used EMG guidance [10,16,20,23] and one paper used ultrasound guidance [21]. The decision on what muscle to inject was made after clinical evaluation. When ECRB was the target, the guidance would have prevented paresis of finger extension [20]. However, even EMG guidance may lead to some leakage of the toxin into adjacent muscles, particularly ECD [10,23].

Decreased grip strength is generally considered a clinical sign of LE, as it is an indirect measurement of pain. Therefore, it serves as an objective parameter of the response to therapy [10]. Finger extensor paresis may interfere with grip strength, as extensor muscles play the role of stabilizers for finger flexors. A transitory reduction in grip strength was recorded with intramuscular BTX-A injection, at 4 and 6 weeks, with further increase at 10, 12, 14, and 16 weeks [12,17,18,19]. Targeting ECRB did not alter the finger extension force or the grip strength [20,26]. With intratendinous injection, grip strength did not differ from the placebo at any moment, although there was a small proportion of patients that reported mild extensor weakness at 4 weeks (33%) and at 12 weeks (7%) when injecting on an anatomical landmark [13]. Using ultrasound guidance, intratendon injection was followed by an increase in grip strength [22].

The type of toxin was aboBTX-A with doses between 40 and 60 UI/injection [12,13,15,22,23], onaBTX-A with doses between 20 and 50 UI/injection or 1 UI/kg, maximum 100 UI/muscle [14,16,18,19], and incoBTX-A with specific doses per muscle [21]. Three papers did not mention the type of BTX-A used for treatment. Comparing different doses of aboBTX-A (10 UI versus 50 UI), the higher dose was more effective. In the literature, the conversion factor for equal potency onaBTX-A:aboBTX-A is ≤1:3 and onaBTX-A:incoBTX-A is 1:1. In the cited papers, the most frequent doses were 50 UI for onaBTX-A and 60 UI for aboBTX-A, a rather high conversion factor.

Three months after a first injection, about 33% of patients may ask for a second injection. Six months after the first injection, 6% of patients may ask for a second and 2% for a third. The cumulative effect of BTX injections offered a rate of success of 80% after one or two administrations [23].

The outcomes of the therapy were pain, grip strength, and function. All papers but one [13] found significative improvement in one or more of the parameters.

A meta-analysis from 2011 underlined the fact that botulinum toxin was more effective than placebo for chronic LE [27,28]. A meta-analysis reported that corticosteroids in LE were more effective in reducing pain in the short term than botulinum toxin, with a higher risk of symptom recurrence and worse outcome [29]. When considering corticosteroid effects on tendon regeneration, botulinum toxin may be considered as an alternative [30,31].

## 5. Conclusions

Lateral epicondylitis resolves spontaneously or with conservative modalities, including oral or local medication, physical agents, therapeutic exercise, and orthosis. However, persistence or recurrence of symptoms may require new therapies or surgical approaches. Botulinum toxin is an option for this purpose, considering that surgery may imply additional risks. Injected either into the tendon or into the muscle, the latter pathway being more successful, botulinum toxin improved clinical and functional status of the patients in this study.

## Figures and Tables

**Table 1:** The main features of the papers cited in the literature.

	Type of Study, No. of Patients	Inclusion/Exclusion Criteria	Injection Point/Targeted Structure	Dose	Outcome	Timing	Results	Side Effects
More, 1997 [10]	Open study/14	Treatment-resistant chronic LE	Electromyographic guidance into extensor fingers III and IV/muscle	20–40 UI BTX-A	Pain	1 month, 3, 6, and 8 months	Pain improvement	Weakness of fingers III and IV extension (expected) for 3–4 months
Keizer, 2002 [11]	Pilot, randomized/40	Pain longer than 6 months, failure of conservative treatment	Anatomical landmark (intramuscular injection ECRB)	30–40 UI BTX-A, 50 UI repeated when necessary/operative (Hohmann procedure)	Pain (VAS) Grip strength Sick leave ROM	Baseline 6 weeks, 3, 6 months, 1 and 2 years	Pain improvement in all groups and moments Sick leave greater at 3 months in operative group ROM reduced at 3 and 6 months in operative group	Patients that did not achieve sufficient paresis had a negative postoperative evolution
Placzek, 2004 [12]	Pilot, prospective, observational/16	Chronic LE, at least 6 months old and failure of 3 therapies	Anatomical landmark: approximately 3 to 4 cm distal to the tender epicondyle, with infiltration of the muscle at two locations; the second location was injected after partial withdrawal of the needle and rotating it in the horizontal plane	60 UI aboBTX	Pain (VAS, clinical pain score) Grip strength	Baseline 2, 6, 10, 14 weeks, 2 years	Pain reduction from the 2nd week, persisting up to 2 years. Grip strength decreased at 2 and 6 weeks and increased at 10 and 14 weeks.	At 2 weeks, there was significant extension weakness in third finger, which disappeared slowly
Wong, 2005 [13]	Randomized, double-blind, placebo-controlled/60	Pain longer than 3 months, no previous injection treatment	Anatomical landmark: deeply into the subcutaneous tissue and muscle, 1 cm from the lateral epicondyle, and aimed toward the tender spot/tendon	60 UI aboBTX/saline (placebo)	Pain (VAS) Grip strength	Baseline 4 weeks 12 weeks	Pain reduction (significant improvement at 4 weeks, maintained at 12 weeks). No diff in grip strength.	Weakness of finger extension and paresis of digits: 33% at 4 weeks, 7% at 12 weeks, with 3% interfering with job activity
Hayton, 2005 [14]	Randomized, double-blinded, placebo-controlled, pilot/40	LE older than 6 months, with failure of therapies	Anatomical landmark: 5 cm distal to the area of maximal tenderness at the lateral epicondyle, in line with the middle of the wrist, the needle inserted deep into the forearm fascia/muscle	50 UI onaBTX-A/saline (placebo)	Pain (VAS) Grip strength General health questionnaire (SF-12)	Baseline 3 months	No significant differences between groups	Extensor lag
Placzek, 2007 [15]	Prospective, placebo-controlled, double-blinded, multicentric/132	Chronic LE; older than 4 months, failure of at least 3 modalities of therapy	Anatomical landmark: approximately 3 to 4 cm distal to the tender epicondyle, with infiltration of the muscle at two locations; the second location was injected after partial withdrawal of the needle and rotating it in the horizontal plane	60 UI aboBTX A/placebo (saline)	Pain (VAS, clinical pain score) Grip strength Subjective assessment	Baseline, 2, 6, 12, and 18 weeks	Pain improved at all moments. Grip strength did not differ at any moment. Subjective assessment improved from week 6.	Weakness of extension of 3rd finger from 2nd week up to 14th week
Oskarson, 2009 [16]	Prospective, observational/10	Chronic pain with failure of previous therapies and surgical reference	ECRB -guided injection under electromyografic stimulation/muscle	1 UI/kg onaBTX-A, maximum 100 UI/muscle/contralateral normal elbow	Pain Function (DASH, COPD) Grip strength Muscle strength ECRB blood flow and lactate concentration	Baseline, 3, 12 months	Blood flow increased at 3 and 12 months. Lactate decreased at 12 months. Pain decreased at 3 and 12 months. Function improved at 12 months. Grip strength declined at 3 months and increased at 12 months.	One patient had abnormal blood flow initially and developed bilateral involvement
Espandar, 2010 [17]	Randomized, placebo-controlled/48	Failure of previous therapies	Anatomical landmark: distance of one-third of the length of the forearm from the tip of the lateral epicondyle on the course of the posterior inter-osseus nerve/muscle	60 UI BTX-A/saline	Pain at rest Pain at maximum grip and pinch Grip strength Extensor lag for 3rd and 4th finger	Baseline 4 weeks 8 weeks 16 weeks	Pain at rest and at maximum pinch decreased significantly. Grip strength decreased transitorily (4 and 8 weeks) but not significantly.	Extensor lag (weakness of extension of digits 3 and 4) was largely present at 4 weeks, resolved at 8 and 16 weeks
Lin, 2010 [18]	Prospective randomized, double-blind, drug-controlled trial/16	Acute and subacute LE	Anatomical landmark: ECRB muscle near common origin of wrist and finger extensors. The needle was first inserted into the subcutaneous layer and then pushed further into the ECRB. Localization of needle tip in the ECRB was confirmed by palpation during resisted wrist extension.	50 UI onaBTX-A/40 mg triamcinolone (CS)	Pain (VAS) Grip strength Quality of life (WHOQOL-BREF)	Baseline 4, 8, 12 weeks	Pain improved significantly in both groups at 4 weeks, better in CS group. Grip strength decreased in BTX (4 and 8 weeks) and increased in CS group. At 12 weeks no significant difference. Quality of life improved in both groups.	Grip strength decreased in BTX group (4 and 8 weeks)
Guo, 2016 [19]	Randomized, prospective, double-blinded, active drug-controlled pilot study/26	LE older than 6 months with failure of physical therapy or oral medication	Anatomical landmark: for the enthesis—1 cm distal to the lateral epicondyle/for the muscles—the most tender point of the common extensor muscles (ECRB or EDC)	20 UI onaBTX-A, 40 mg triamcinolone (CS)	Pain (VAS) Grip strength PRTEE	Baseline 4, 8, 12, and 16 weeks	At 4 weeks: intratendon BTX-A and CS had better results than intramuscular BTX-A. At 8, 12, and 16 weeks—no difference.	Intramuscular: extensor lag with full recovery Grip strength transitory reduced at 4 weeks for intramuscular BTX
Creuze, 2018 [20]	Phase-III, single-center, randomized, double-blinded, placebo-controlled/60	Chronic LE 6 months old Failure of previous therapies	Anatomical landmark: at approximately 5 cm distal to the lateral epicondyle (targeting ECRB), EMG confirmation	40 UI aboBTX-A/saline	Pain (VAS) Grip strength Quality of life (self-assessment)	Baseline 30 and 90 days	Pain improved at both moments No difference for grip strength	No extensor lag No grip strength alteration
Ruiz, 2019 [21]	Prospective, experimental/24	Chronic pain with failure of previous therapies	Ultrasound-guided infiltration into specific muscle	Specific doses incoBTX-A per muscle, maximum 80 UI	Pain (VAS) Function (QuickDASH)	Baseline 1, 3, and 6 months	Pain and function improved at 1 month and persisted at 6 months	21% failure 13% required a second dose (positive effect, short duration)
Lee, 2019 [22]	Prospective, randomized, comparative/60	Pain longer than 3 months Failure of previous therapies	Ultrasound guidance: peppering technique in the tendon in a distal-to-proximal direction	10 UI aboBTX-A (SD)/50 UI BTX-A (LD)	Pain (NRS) Grip strength Motor weakness in extensors	Baseline 1, 2, 3, 4, 5, and 6 months	Pain decreased in both groups at all moments, LD group had better results at all moments. Grip strength increase in both groups, all moments, better results in LD.	Extensor weakness 3% in SD and 20% in LD
Cogne, 2019 [23]	Open, prospective, observation, continuation of Creuze, 2018 (19)/50	Follow-up after first BTX-A injection	Anatomical landmark: at approximately 5 cm distal to the lateral epicondyle (targeting ECRB), EMG confirmation	40 UI aboBTX-A	Number of required injections	270 and 365 days	80% of patients improved after 1 or 2 injections 2% asked for a third injection	18% of patients asked for surgery

VAS, visual analogue scale; ROM, range of motion; NRS, numeric rate scale; DASH, disability of arm, shoulder, and hand; ECRB, extensor carpi radialis brevis muscle; COPD, Canadian Occupational Performance Measure; PRTEE, patient-rated tennis elbow evaluation; WHOQOL-BREF, World Health Organization Quality of Life Brief Questionnaire—BREF; CS, corticosteroid.

## Data Availability

All data are available online in the mentioned databases.
